# Adrenal Abcg1 Controls Cholesterol Flux and Steroidogenesis

**DOI:** 10.1210/endocr/bqae014

**Published:** 2024-02-01

**Authors:** Jani Liimatta, Evelyn Curschellas, Emre Murat Altinkilic, Rawda Naamneh Elzenaty, Philipp Augsburger, Therina du Toit, Clarissa D Voegel, David T Breault, Christa E Flück, Emanuele Pignatti

**Affiliations:** Division of Pediatric Endocrinology, Diabetology and Metabolism, Department of Pediatrics, Inselspital, Bern University Hospital, Bern 3010, Switzerland; Department for BioMedical Research, University Hospital Inselspital, University of Bern, Bern 3010, Switzerland; Kuopio Pediatric Research Unit (KuPRU), University of Eastern Finland and Kuopio University Hospital, Kuopio 70200, Finland; Department of Chemistry, Biochemistry and Pharmacy, Medical Faculty, University of Bern, Bern 3010, Switzerland; Division of Pediatric Endocrinology, Diabetology and Metabolism, Department of Pediatrics, Inselspital, Bern University Hospital, Bern 3010, Switzerland; Department for BioMedical Research, University Hospital Inselspital, University of Bern, Bern 3010, Switzerland; Department for BioMedical Research, University Hospital Inselspital, University of Bern, Bern 3010, Switzerland; Department for BioMedical Research, University Hospital Inselspital, University of Bern, Bern 3010, Switzerland; Division of Pediatric Endocrinology, Diabetology and Metabolism, Department of Pediatrics, Inselspital, Bern University Hospital, Bern 3010, Switzerland; Department for BioMedical Research, University Hospital Inselspital, University of Bern, Bern 3010, Switzerland; Department of Nephrology and Hypertension, Inselspital, Bern University Hospital, University of Bern, Bern 3010, Switzerland; Department for BioMedical Research, University Hospital Inselspital, University of Bern, Bern 3010, Switzerland; Department of Nephrology and Hypertension, Inselspital, Bern University Hospital, University of Bern, Bern 3010, Switzerland; Department of Pediatrics, Harvard Medical School, Boston Children's Hospital, Boston, MA 02115, USA; Harvard Stem Cell Institute, Cambridge, MA 02138, USA; Division of Pediatric Endocrinology, Diabetology and Metabolism, Department of Pediatrics, Inselspital, Bern University Hospital, Bern 3010, Switzerland; Department for BioMedical Research, University Hospital Inselspital, University of Bern, Bern 3010, Switzerland; Division of Pediatric Endocrinology, Diabetology and Metabolism, Department of Pediatrics, Inselspital, Bern University Hospital, Bern 3010, Switzerland; Department for BioMedical Research, University Hospital Inselspital, University of Bern, Bern 3010, Switzerland

**Keywords:** Abcg1, cholesterol, glucocorticoids, steroids, adrenal cortex

## Abstract

Cholesterol is the precursor of all steroids, but how cholesterol flux is controlled in steroidogenic tissues is poorly understood. The cholesterol exporter ABCG1 is an essential component of the reverse cholesterol pathway and its global inactivation results in neutral lipid redistribution to tissue macrophages. The function of ABCG1 in steroidogenic tissues, however, has not been explored. To model this, we inactivated Abcg1 in the mouse adrenal cortex, which led to an adrenal-specific increase in transcripts involved in cholesterol uptake and de novo synthesis. Abcg1 inactivation did not affect adrenal cholesterol content, zonation, or serum lipid profile. Instead, we observed a moderate increase in corticosterone production that was not recapitulated by the inactivation of the functionally similar cholesterol exporter Abca1. Altogether, our data imply that Abcg1 controls cholesterol uptake and biosynthesis and regulates glucocorticoid production in the adrenal cortex, introducing the possibility that *ABCG1* variants may account for physiological or subclinical variation in stress response.

Steroid hormones mediate a myriad of physiological responses, from the control of blood pressure (mineralocorticoids) and sexual maturation (sex hormones) to the regulation of glucose homeostasis and stress response (glucocorticoids) (1). The extensive impact of steroid hormones on human physiology demands a fine regulation of steroid production. Alterations of this fine balance may result in pathological phenotypes, including adrenocortical insufficiency or steroid hypersecretion, for which many monogenic or polygenic determinants still need to be identified (2, 3).

As the obligatory precursor of all steroids, cholesterol is a key modulator of steroidogenesis, both in a quantitative and qualitative fashion. Disruption of cholesterol homeostasis results in adrenal insufficiency (eg, in Smith-Lemli-Opitz disease) (4), while the dysregulated accumulation of cholesterol leads to increased cholesterol storage and physical and biochemical cellular distress (eg, in lipoid congenital adrenal hyperplasia) (5-7). Besides, fine tuning of cholesterol homeostasis is critical for regulation of steroidogenesis within a physiological range. For instance, interfering with cholesterol content in plasma membranes directly impacts the synthesis of pregnenolone, which is a common precursor to all steroids (8). In addition, the master transcriptional activator of steroidogenesis, Steroidogenic Factor 1 (NR5A1), not only induces the expression of critical steroidogenic enzymes but also triggers the expression of cholesterogenic genes to provide more substrate for steroidogenesis (9). Furthermore, our group previously showed that intracellular cholesterol deprivation reroutes steroidogenesis to a more androgenic profile, implicating cholesterol in the prioritization of steroidogenic pathways (10).

Levels of intracellular cholesterol are therefore finely balanced between cholesterol acquisition (contributed by uptake from the circulation, de novo biosynthesis, and hydrolysis of cholesteryl esters) and disposal (mediated by excretion of cholesterol to the circulation, cholesterol esterification for long-term storage, and cholesterol deployment for biosynthesis of downstream products) (11). Intracellular cholesterol homeostasis in adrenocortical cells is thought to rely on the sterol regulatory element-binding factor 2, which acts as a master transcriptional activator of the cholesterol biosynthetic pathway and the cholesterol import machinery upon conditions of sterol depletion (7, 11-13). However, the molecular programs that control cholesterol availability in steroidogenic cells are not fully characterized.

Abcg1 is an adenosine triphosphate-dependent transporter involved in the maintenance of tissue and cellular cholesterol homeostasis. In mice and humans, it is expressed in a variety of cell types including adrenocortical cells (14-22). Its subcellular localization is still a matter of debate: Abcg1 has been found in both endosomes and in the plasma membrane, and in association with actin filaments (23-27). It is thought to mobilize cholesterol from the endoplasmic reticulum and to redistribute it to the plasma membrane, favoring cholesterol efflux to a variety of extracellular acceptors (15, 28-30). The role of Abcg1 in steroidogenic tissues, however, is unknown.

Here we study the adrenal cortex to determine the role of Abcg1 in a steroidogenic tissue. Abcg1 inactivation in mouse adrenals results in increased transcripts for cholesterol biosynthesis and uptake, leading to increased corticosterone production. Our data suggest that Abcg1 is a key regulator of cholesterol flux and glucocorticoid production.

## Methods

### Experimental Animals

All animal procedures were approved by the Veterinary Office of the Canton Bern in Switzerland. Generation of the aldosterone synthase-Cre strain (Cyp11b2^tm1.1(cre)Brlt^), and the compound conditional *Abcg1* and *Abca1* strain (B6.Cg-*Abca1^tm1Jp^ Abcg1^tm1Tall^*^/J^), was previously described (31, 32). To generate the bigenic mice carrying 1 Cre allele and 2 conditional alleles either within the *Abca1* or the *Abcg1* locus (referred to as *Abca1 cKO* and *Abcg1 cKO*, respectively), males of the Cre-bearing strain were crossed with compound heterozygous females for the conditional *Abca1* and *Abcg1* alleles. Pups expressing the Cre recombinase and either the *Abca1^tm1Jp^* or the *Abcg1^tm1Tall^*^/J^ allele were selected and crossed with isogenic littermates. Littermates carrying the Cre allele alone, or 1 of the 2 conditional alleles, were used as controls. All mice were kept on a mixed sv129-C57BL/6 genetic background, with free access to chow and water, under a 12-hour light/12-hour dark cycle. Unless otherwise specified, all mice used for this work were 2-month-old females. Adrenal weight was measured on an analytical balance on freshly dissected adrenal glands following clearance of the surrounding fat tissue.

### Gene Expression Analysis

RNA was isolated from whole adrenals cleaned of the adherent fat or from livers and homogenized in TRI Reagent (Sigma) using the Direct-zol miniprep RNA kit (Zymo Research), following the manufacturer's protocol. RNA was reverse transcribed using the High-Capacity cDNA Reverse Transcription Kit (Thermo Fisher Scientific). Gene expression analysis was performed by real-time quantitative PCR using the PowerUp SYBR Master Mix and the QuantStudio 1 thermocycler (Thermo Fisher Scientific). Technical duplicates were used to minimize variability. For mouse studies, the following primers were used: *Abcg1*, Fw: ACATCGAATTCAAGGACCTT, Rv: CCCAGAGATCCCTTTCAAAA; *Abca1*, Fw: AACTTTCAAGATGCTGACTG, Rv: AAAGAACTCCACATGCTCTC; *Ldlr*, Fw: GTTGCAGCAGAAGACTCAT, Rv: CACCCACTTGCTAGCGAT; *Scarb1,* Fw: CAGGTGCTCAAGAATGTCC, Rv: TAGAAAGGGACGGGGATC; *Hmgcr*, Fw: AATGCCTTGTGATTGGAGTT, Rv: CCGGGAAGAATGTCATGAA; *Sqle,* Fw: AAAGAAAGAACAGCTGGAGT, Rv: TAGCTGCTCCTGTTAATGTC; *Insig1,* Fw: ATAGCCACCATCTTCTCCTC, Rv: TCTCTCTTGAACTTGTGTGG; *Gck*, Fw: TGTAAGGCACGAAGACATAG, Rv: GTTGTTCCCTTCTGCTCC; *Pck1*, Fw: GTGGAAGGTCGAATGTGTG, Rv: TTGATAGCCCTTAAGTTGCC; *G6pc*, Fw: GTTCAACCTCGTCTTCAAGT Rv: CTGTTGCTGTAGTAGTCGG; *Nr3c1*, Fw: CTATGAACTTCGCAGGCC Rv: GAGAACTCACATCTGGTCTC; *Gapdh*, Fw: ATCAACGACCCCTTCATTG, Rv: TTGATGACAAGCTTCCCATT; *Actb*, Fw: GACCTGACAGACTACCTCAT, Rv: CTCGAAGTCTAGAGCAACAT. Transcripts encoding glyceraldehyde 3-phosphate dehydrogenase or actin beta were used as internal control, and data were expressed using the 2^−ddCt^ method.

### Transcriptome Profiling

Preparation of whole-adrenal RNA isolates was conducted as indicated in the Gene Expression Analysis section. RNA samples were quantified using the RiboGreen assay (Thermo Fisher Scientific). Sample quality was analyzed on a Fragment Analyzer 5200 (Agilent) using the Fragment Analyzer HS RNA kit(15NT) (Agilent, DNF-472-1000). Illumina Stranded mRNA Prep Preparation including polyA enrichment was used according to the manufacturer's recommendations to construct libraries from total RNA. Subsequently, the Illumina NovaSeq and NextSeq platforms with a NovaSeq 200cy Kit (v1.5) and a NextSeq 300cy Kit (v2), respectively, were used to sequence the libraries. The produced paired-end reads that passed Illumina's chastity filter were demultiplexed using Illumina's bcl2fastq software version 2.20.0.422 (no further refinement or selection). Illumina adapter residuals were trimmed using cutadapt (v4.0 with Python 3.9.16). Quality of the reads in fastq format was checked with the software FastQC (version 0.11.9). Raw reads shorter than 10 bp, having average Q-values below 24, or incorporating uncalled “N” bases were filtered out using the BBTools software suite (version 38.86). The splice-aware RNA mapping software STAR (version 2.7.10a) was used to map the remaining reads to the mm10 reference genome provided by IGenomes (archive-2015-07-17-14-33-26). To count the uniquely mapped reads to annotated genes, the software htseq-count (HTSeq version 0.13.5) was used. Normalization of the raw counts and differential gene expression analysis was carried out with the R software package DESeq2 (version 1.38.3). Combined evidence from previous works suggest that only about 50% to 60% of cells contributing to whole-adrenal transcriptome profiling efforts are recombined steroidogenic cells of interest (31, 33). Therefore, we expected differentially expressed genes to be less abundant in our whole-adrenal extracts with respect to more enriched cell populations (eg, sorted cortical cells) and used a relaxed fold change threshold (1.2) to capture these genes. Library construction, sequencing, and data analysis described in this section were performed by Microsynth AG (Balgach, Switzerland). Profiling results are stored within the Gene Expression Omnibus repository under the accession number GSE242081.

### Histology, Immunofluorescence, and Microscopy

Adrenals were dissected, cleared of the fat tissue, and fixed overnight in 4% paraformaldehyde. Four-µm paraffin sections were processed for protein immunodetection as previously described (10). Briefly, antigen retrieval was performed in 10 mM Sodium Citrate pH 6, and incubation was conducted overnight using a mouse monoclonal anti-Disabled-2/p96 (Dab2; BD Transduction Laboratories, cat. no. 610464; RRID: AB_397837) and a rabbit polyclonal anti-Akr1b7 [kindly provided by Dr Pierre Val and Dr Antoine Martinez; RRID: AB_3075891 (34)]. Indirect staining was performed using the goat anti-rabbit IgG (H + L) highly cross-adsorbed secondary antibody conjugated with Alexa Fluor™ 488 (from Thermo Fisher Scientific, cat. no. A11008; RRID: AB_143165), and a goat anti-mouse IgG (H + L) cross-adsorbed secondary antibody conjugated with Alexa Fluor™ 647 (from Thermo Fisher Scientific, cat. no. A21235; RRID: AB_2535804). 4′,6-diamidino-2-phenylindole was used for counterstaining. Images were captured with a Nikon Eclipse Ti-E microscope. Hematoxylin and eosin staining was carried out on neighboring sections compared to the immunofluorescence experiment. For Oil Red O staining, mouse adrenals were snap frozen and 5-µm sections were processed in a mixed solution of Oil Red O and dextrin, followed by nuclear counterstaining with Mayer's hemalum (all products from Merck).

### Steroid Profiling and Blood Tests

Mouse serum was obtained using cardiac puncture of mice euthanized by intraperitoneal injection of pentobarbital. This terminal procedure was chosen because it allowed the collection of paired blood and adrenal tissue samples while causing a significantly lower stress response in mice compared to other euthanasia methods (35). Twenty-five  µL of serum was used for further liquid chromatography-mass spectrometry (LC-MS) analysis using an established in house LC-MS method (36). Briefly, samples were collected and stored at −20 °C. Following thawing, 38 µL of internal standard was added to 25 µL of sample and extracted with ZnSO_4_ and methanol. After centrifugation, the organic phase was purified using a solid-phase extraction on an OasisPrime HLB 96-well plate using a positive pressure 96-well processor (both Waters, UK). For LC-MS analysis, a Vanquish UHPLC (equipped with an ACQUITY UPLC HSS T3 Column, 100 Å, 1.8 µm, 1 mm × 100 mm column; Waters, Switzerland) was coupled to a Q Exactive Plus Orbitrap (both Thermo Fisher Scientific, Reinach, Switzerland). Separation was achieved using gradient elution over 17 minutes using water and methanol both supplemented with 0.1% formic acid (all Sigma-Aldrich, Buchs, Switzerland) as mobile phases. Data analysis was performed using TraceFinder 4.1 (Thermo Fisher Scientific). The method was validated according to international standards. Steroid hormone concentrations were calculated in nmol/L. Values detected below the lower limit of accurate quantification were not used for statistics. Adrenocorticotropin hormone (ACTH) in serum was measured using an enzyme-linked immunosorbent assay kit (Abcam, cat. no. ab263880; RRID: AB_2910221), following the manufacturer's protocol. While ACTH is routinely assayed in plasma, we preferred quantification in serum as suggested by the kit based on the equivalence of serum and plasma for ACTH measurement in humans (37). Total cholesterol, high-density lipoproteins (HDL), and low-density lipoproteins/very-low density lipoproteins particles were measured using a cholesterol assay kit (Abcam, cat. no. ab65390), while triglycerides were assayed with a triglyceride assay kit (Abcam, cat. no. ab65336), following the manufacturer's instructions.

### In Situ Hybridization

For double enzymatic in situ hybridization, mice adrenal glands were fixed in 4% paraformaldehyde at 4 °C for 24 hours and 5-μm-thick sections from Formalin-Fixed Paraffin-Embedded blocks were cut. In situ hybridization was performed following the manufacturer's recommendation of the BaseScope Duplex Reagent Kit Intro Pack-Mm (Advanced Cell Diagnostics, cat. no. 323871). Standard conditions were used: 15 minutes incubation for the Antigen retrieval step and 30 minutes for Protease III treatment. In situ hybridization staining was performed manually with the following combinations of RNAscope® probes (all from Bio-Techne). BA-Mm-Abca1-3ZZ-st-C2 probe, recognizing Abca1 (cat no. 1218611-C2) detected with the Fast Red signal; BA-Mm-Abcg-E3-1ZZ-st-C1 probe, recognizing Abcg1, (cat no. 1218601-C1) detected with the green signal. Basescope Duplex Positive Control Probe-Mouse(Mm)-C1-Ppib-1ZZ/C2-Polr2a-3ZZ and Basescope Duplex Negative Control Probe-C1-DapB-3ZZ/C2-DapB-3ZZ (cat no. 322982) were used respectively as positive and negative controls. Nuclei were visualized using hematoxylin, and slides were mounted with Vectamount mounting medium (Vector Labs, cat. no. H5000). Images were acquired on a NanoZoomer S60 digital slide scanner at 40× (Hamamatsu).

### Cholesterol Quantification

Adrenal glands were dissected, clear of the surrounding fat pad, and homogenized in radioimmunoprecipitation assay buffer (Pierce, cat. no. 89900) supplemented with a protease and phosphatase inhibitor by Thermo Scientific (cat. no. A32961) at 4 °C, using lysing matrix tubes (MP Biomedicals, cat. no. 6913100) and a Bead Mill Homogenizer by Omni International. Tissue lysates were incubated for 1 hour in ice and spun down on a bench centrifuge for 20 minutes at 4 °C to get rid of unprocessed debris. Quantification of cholesterol was carried out using a Cholesterol/Cholesterol Ester-Glo^TM^ Assay by Promega (cat. no. J3190) following the manufacturer's instructions, with the exception that adrenal lysates were diluted from 1:10 to 1:40 in the lysis buffer provided by the kit to fit the calibration curve. The assay was performed either with or without cholesterol esterase, to allow for quantification of both total and free cholesterol, respectively. Values for esterified cholesterol were obtained by subtraction of free from total cholesterol. All cholesterol values were normalized by protein concentration assayed using a DC protein assay (Bio-Rad, cat. no. 5000112).

### Statistical Analysis

Two-tailed Student's t-test was used for comparisons between any 2 groups. For every comparison, the F-test was used to assess inequality of variances. In case of inequality of variances, the Welch correction was adopted. One-way ANOVA and Dunnett’s multiple comparison test were used for comparisons between groups of 3 or more, unless otherwise specified. Prism 10 software (GraphPad) was used for statistical analysis. All data were included; no exclusion method was applied. Data are presented as mean ± SEM.

## Results

### Loss of Adrenocortical Abcg1 Increases Transcripts Involved in Cholesterol Metabolism

**Figure 1.**
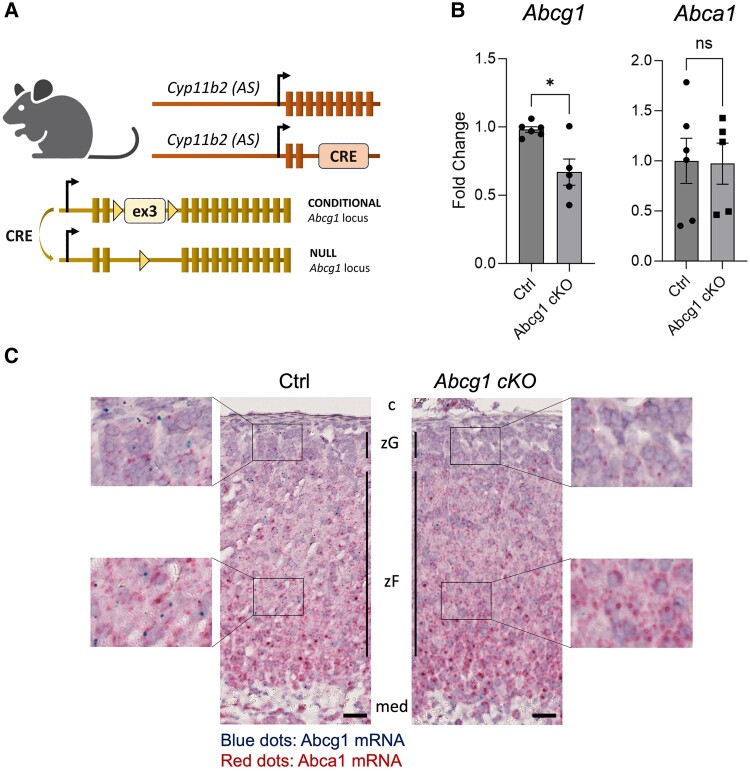
Effective gene recombination in *Abcg1 cKO* mice. (A) Schematic representation of the mouse model used to inactivate *Abcg1* in adrenocortical cells using Cre-mediated recombination of *Abcg1*'s third exon. (B) Quantitation of transcripts encoding Abcg1 and Abca1 in control and *Abcg1 cKO* adrenal glands. (C) In situ depiction of *Abcg1* (blue dots) and *Abca1* (red dots) transcripts in control (left) and *Abcg1 cKO* adrenal sections (right), including insets’ virtual magnifications on each side. All mice used for this figure were 2-month-old females. Scale bar = 25μm. *P ≤ .05.

Abbreviations: AS, aldosterone synthase; c, capsule; med, medulla; ns, not significant; zF, zona Fasciculata; zG, zona Glomerulosa.

To investigate the role of Abcg1 in the adrenal cortex, we generated a mouse model where both *Abcg1* alleles were conditionally inactivated using an aldosterone synthase (*Cyp11b2*)-specific Cre recombinase (Fig. 1A). The efficiency and extent of recombination were determined by quantifying *Abcg1* transcripts within control and conditional knockout adrenals (henceforth referred to as *Abcg1 cKO*), and by in situ visualization of *Abcg1* mRNAs. Specifically, *Abcg1* transcripts were reduced by about 40% in recombined whole adrenals (Fig. 1B), and recombination occurred throughout the entire cortex (Fig. 1C). Importantly, transcripts encoding Abca1, a functionally similar adenosine triphosphate-dependent cholesterol exporter (11), were not affected by *Abcg1* knockout (Fig. 1B).

**Figure 2.**
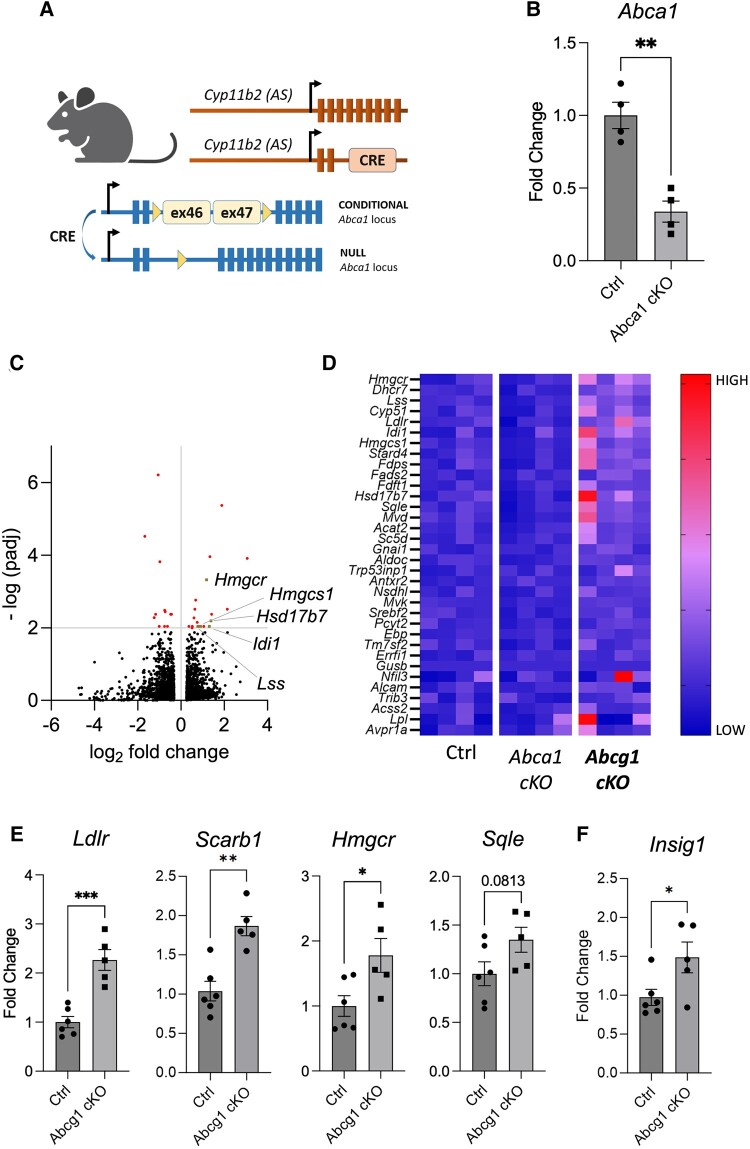
Inactivation of adrenocortical *Abcg1* results in increased transcripts for cholesterol uptake and synthesis. (A) Schematic representation of the mouse model used to inactivate *Abca1* in adrenocortical cells using Cre-mediated recombination of exons 46 and 47. (B) Quantification of *Abca1* transcripts in control and *Abca1 cKO* adrenal glands. (C) Volcano plot depicting 12 downregulated and 19 upregulated genes (red or beige dots) in *Abcg1 cKO* adrenals compared to the combined (summed) datasets of controls and *Abca1 cKO* counterparts, using cutoffs of 1.2 for fold change and 0.01 for adjusted *P*-value. Each beige dot is associated with a gene name as indicated in the plot. (D) Heat map depicting color-coded expression levels of 34 transcripts responsible for the enrichment of the HALLMARK_CHOLESTEROL_HOMEOSTASIS dataset in Gene Set Enrichment Analysis. (E and F) Quantitation of transcripts involved in cholesterol regulation, uptake, and de novo synthesis in control and *Abcg1 cKO* adrenal glands. All mice used for this figure were 2-month-old females. *P ≤ .05; **P ≤ .01; ***P ≤ .001.

Abbreviation: AS, aldosterone synthase.

To assess the impact of *Abcg1* on adrenal physiology, we profiled the transcriptome of *Abcg1* cKO adrenals and compared it with the transcriptome of control and *Abca1* cKO counterparts, which were also used as controls (Fig. 2A and 2B). Using a cutoff of 1.2 for fold change and 0.01 for adjusted *P*-value, we found 19 upregulated and 12 downregulated genes specifically in *Abcg1* knockout adrenal glands (Fig. 2C). Gene set enrichment analysis revealed that cholesterol metabolism was the most affected pathway, with 34 genes contributing to the cholesterol set enrichment (HALLMARK_CHOLESTEROL_HOMEOSTASIS dataset) (Fig. 2D). Using quantitative PCR, we validated 3 of these upregulated genes, either implicated in cholesterol uptake (*Ldlr*) or biosynthesis (*Hmgcr*, *Sqle*) (Fig. 2E). The gene encoding the HDL receptor (*Scarb1*), which is the main route for cholesterol delivery to steroidogenic pathways (38), resulted upregulated using quantitative PCR (Fig. 2E), despite not contributing to the enrichment of the gene set enrichment analysis dataset (Fig. 2C and 2D). To determine the adrenal perception of cholesterol load, we also quantified *Insig1*, which is normally reduced upon accumulation of sterols (39, 40). Surprisingly, we found that *Insig1* was upregulated in *Abcg1 cKO* adrenals (Fig. 2F), suggesting that cholesterol metabolism in *Abcg1*-deficient glands is dysregulated.

Altogether, our data indicate that Abcg1 deficiency in the adrenal cortex disrupts intracellular cholesterol homeostasis by driving the expression of transcripts that normally promote increased cholesterol production and uptake.

### Loss of Abcg1 Results in Increased Corticosterone

**Figure 3.**
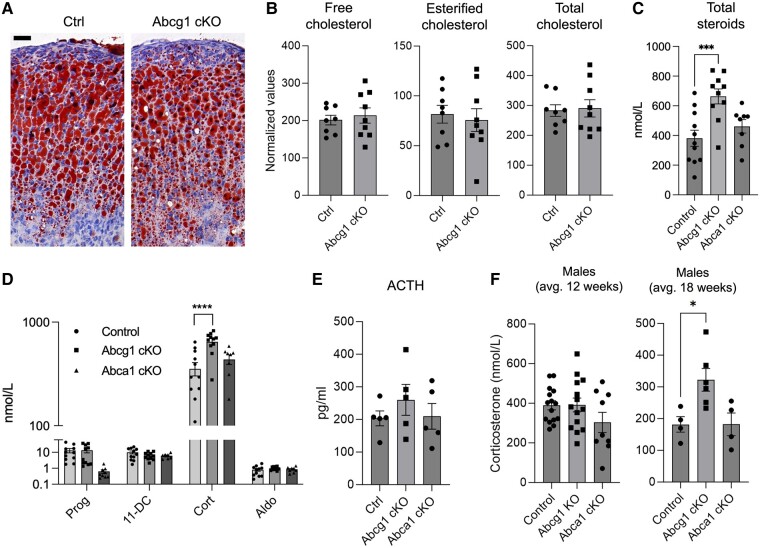
Inactivation of adrenocortical *Abcg1* results in increased corticosterone synthesis. (A) Oil Red O staining (red) of control and *Abcg1 cKO* adrenocortical sections. Mayer's hemalum was used to counterstain nuclei (blue). Images are representative of 4 animals per genotype. Scale bar = 25 µm. (B) Free, esterified, and total cholesterol in whole adrenal glands from control and *Abcg1 cKO* animals. (C) Aggregated quantification of adrenal steroids detected in mouse sera using liquid chromatography-mass spectrometry, ie, pregnenolone, Prog, 11-DC, Cort, and Aldo. (D) Steroid concentrations in sera of control and *Abcg1 cKO* mice. Most pregnenolone values were below the threshold of accurate quantification, likely because of intense processivity into downstream products, and are not reported in this graph. (E) Levels in sera from control and *Abcg1 cKO* mice. (F) Levels of corticosterone in male control and *Abcg1 cKO* mice at different ages. Except for panel F, all mice used for this figure were 2-month-old females. *P ≤ .05, ***P ≤ .001, ****P ≤ .0001.

Abbreviations: 11-DC, 11-deoxycorticosterone; ACTH, adrenocorticotropin hormone; Aldo, aldosterone; avg, average; Cort, corticosterone; Prog, progesterone.

To determine whether increased cholesterol-related transcripts driven by *Abcg1* inactivation results in increased cholesterol storage, we performed an Oil Red O staining of adrenal sections and observed no difference between *Abcg1 cKO* and control tissues (Fig. 3A). Direct quantification of total, free, and esterified cholesterol in the adrenals confirmed that cellular cholesterol compartments are not impacted by inactivation of Abcg1 (Fig. 3B).

We then investigated whether the increase in cholesterol-related transcripts might lead to an increase of steroid biosynthesis. The adrenal steroid output (ie, the sum of pregnenolone, progesterone, 11-deoxycorticosterone, corticosterone, and aldosterone) showed a 74% increase in *Abcg1 cKO* mice compared to control animals. Instead, *Abca1 cKO* mice did not display any change in adrenal steroid metabolites (Fig. 3C). Most of the variation in *Abcg1 cKO* steroid profile was explained by increased corticosterone, the main glucocorticoid in mice, whereas the other steroids were not affected (Fig. 3D). The increase in corticosterone, although significant, was not sufficient to suppress the level of its main secretagogue, ACTH (Fig. 3E). While these results are based on female mice, male counterparts displayed a comparable increase in corticosterone but at an older age (average 18 weeks for males, compared with 12 weeks for females) (Fig. 3D and 3F).

**Figure 4.**
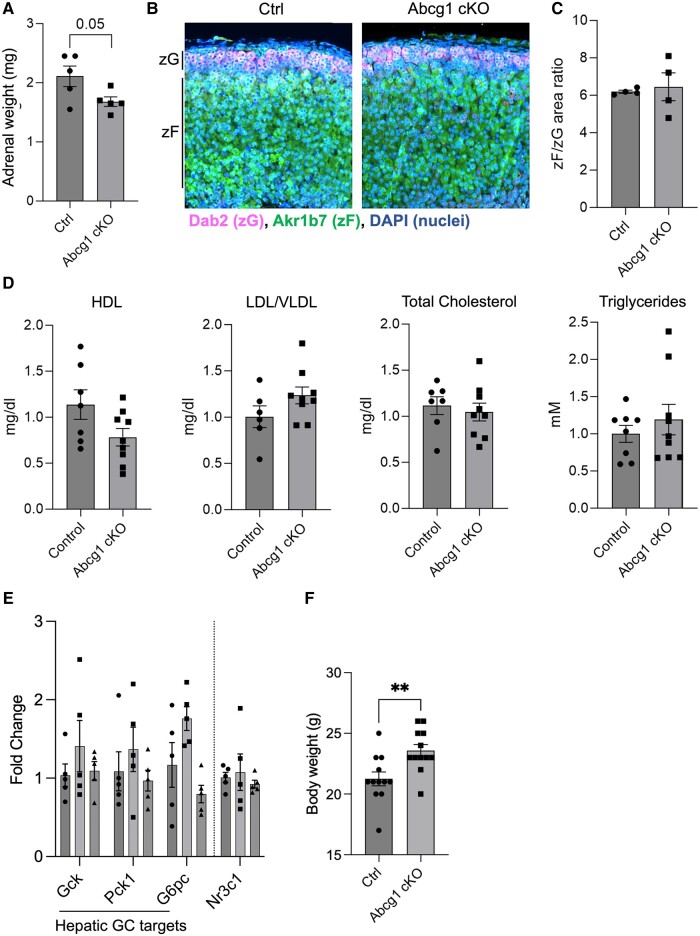
*Abcg1 cKO* mice display increased body weight but unaltered adrenal mass, zonation, and serum lipid profile. (A) Adrenal weight measured on freshly dissected whole adrenals in control and *Abcg1 cKO* mice. (B) Representative depiction of immunofluorescence assay on adrenocortical sections from control and *Abcg1 cKO* mice. Images are representative of 4 animals per genotype. Scale bar = 50 µm. (C) Ratio of the zF area (measured as the area stained by Akr1b7) and the zG area (measured as the area stained by Dab2). (D) Lipid profile in control and *Abcg1 cKO* mouse sera. (E). Quantification of glucocorticoid-sensitive (ie, *Gck*, *Pck1*, *G6pc*) and insensitive (*Nr3c1*) genes in livers from control, *Abcg1 cKO*, and *Abca1 cKO* animals. (F) Quantification of live animal weight. All mice used for this figure were 2-month-old females. **P ≤ .01.

Abbreviations: GC, glucocorticoids; zF, zona Fasciculata; zG, zona Glomerulosa

We then evaluated whether the increase in corticosterone was associated with increased adrenal size or altered zonation. First, we assessed adrenal weight, which revealed *Abcg1 cKO* adrenals mice were unchanged, compared to controls, with a paradoxical trend toward a decrease in adrenal weight (Fig. 4A). Next, we stained for the zone-specific markers Dab2 [identifying the zona Glomerulosa (zG)] and Akr1b7 [identifying the zona Fasciculata (zF)], which showed no difference between control and *Abcg1* cKO mice in the zF-to-zG area ratio (Fig. 4B and 4C). These results indicate that neither increased adrenal mass nor expansion of the zF explains the increased corticosterone production in *Abcg1 cKO* mice.

Furthermore, to exclude that corticosterone production was influenced by a change in systemic lipid metabolism in *Abcg1 cKO* mice, we performed serum lipid profiling, which revealed no differences in HDL, low-density lipoproteins, total cholesterol, or triglycerides between *Abcg1 cKO* and control mice (Fig. 4D).

Finally, the systemic response to increased glucocorticoid was estimated in *Abcg1 cKO* mice by quantifying 3 glucocorticoid target genes in the liver, ie, *Gck*, *Pck1*, and *G6pc* (41-44), which showed a nonsignificant trend of increase compared to control and *Abca1 cKO* animals (Fig. 4E). Instead, no such trend was observed for *Nr3c1*, whose expression levels are not sensitive to circulating glucocorticoids (Fig. 4E) (44). In addition, *Abcg1 cKO* mice displayed a mild increase in body weight compared to control animals (Fig. 4F), compatible with a moderate but prolonged exposure to increased corticosterone (45).

Altogether, our data suggest that loss of *Abcg1* results in increased intracellular cholesterol uptake and biosynthesis, leading to higher glucocorticoid production.

## Discussion

We show that inactivation of Abcg1 in the adrenal cortex leads to increased expression of genes that promote cholesterol availability (from uptake and biosynthesis), as well as an increase in glucocorticoid production. The increase in glucocorticoid production was observed in both female and male mice, albeit at an older age in male mice, possibly due to a slower rate of recombination and/or tissue turnover in these mice (46, 47).

Although our work does not provide an integrated analysis of 24-hour urine corticosterone metabolites, the absence of ACTH suppression and the analysis of corticosterone-responsive liver genes suggest that *Abcg1 cKO* mice show only a mild increase of daily corticosterone output, most likely within physiological range. Consistent with this conclusion, we expect only a minor (if any) impact on glucose metabolism, which was not directly investigated in this work. The increase in body weight in *Abcg1 cKO* mice is compatible with a protracted exposure to moderately increased corticosterone levels (45). In addition, the ACTH values averaging 200 pg/mL throughout all our animal groups possibly reflect a mild stress stimulation, compatible with reported values in rats upon pentobarbital-mediated terminal anesthesia (48).

Surprisingly, our data are in contrast with the mild glucocorticoid insufficiency and decreased cortical cholesteryl esters found by Hoekstra and colleagues in mice following global deletion of *Abcg1* (20). This discrepancy could be explained by a possible decrease in corticotropin releasing hormone and/or ACTH in mice with global *Abcg1* deletion, which were not assayed in the study. Alternatively, global loss of *Abcg1* could lead to functional impairment or dysgenesis of the adrenal cortex, underlying a not yet described role of *Abcg1* during intrauterine development. This latter hypothesis is less plausible, though, because of the low level of *ABCG1* expression reported in human fetal tissues (21). In our work, we use a conditional mouse model that leads to inactivation of *Abcg1* specifically in the steroidogenic cells of the adrenal cortex during the first weeks of postnatal development (31), which allows us to rule out prenatal or systemic effects of *Abcg1* deletion on the phenotype. However, an accurate quantification of the extent of recombination in *Abcg1 cKO* adrenals is technically challenging. Therefore, we cannot exclude the possibility that the differences between Hoekstra and olleagues’ work (20) and ours are due to a different degree of *Abcg1* recombination in adrenocortical cells.

Our finding that adrenal *Abcg1* inactivation results in upregulation of transcripts important for cholesterol biosynthesis and uptake is in line with the increases seen in *Hmgcr*, Farnesyl pyrophosphate (*Fpp*), and *Ldlr* in the liver from global *Abcg1 KO* mice (15). This similarity suggests that the genetic network regulated by *Abcg1* is conserved among different tissues.

Abcg1 inactivation, however, did not affect transcripts encoding genes directly implicated in steroidogenic conversions, raising the hypothesis that increased adrenal steroidogenesis might be due to excess cholesterol in *Abcg1 cKO* mice flowing directly into the steroidogenic machinery and fueling the production of the end-product corticosterone. This hypothesis implies that the amounts of cholesterol entering the steroidogenic pathway are loosely controlled, and exposure to functional cholesterol sources (eg, lipoproteins) may directly trigger increased steroidogenesis. While, to our knowledge, this has not been formally tested in vivo, steroidogenesis is directly stimulated by exposure to lipoproteins in primary adrenal cells and in the established NCI-H295R adrenal cell line (49) (and our data, not shown).

It is interesting to note that aldosterone, despite being an adrenal functional end-product, is not affected by Abcg1 inactivation. This is surprising in consideration of the fact that exposure to cholesterol results in increased aldosterone production in vitro (49, 50). We suspect this difference is because aldosterone synthase (Cyp11b2) expression, unlike the expression of 11-beta-hydroxylase (Cyp11b1—the last step in corticosterone biosynthesis) is finely tuned in mice by a range of physiological stimuli. In fact, the expression of Cyp11b2 in mice and rats, unlike in cells, is regulated in such a way that only a subset of zG cells express the enzyme at a given time (51). Excess sodium can suppress Cyp11b2 expression almost completely, while poor dietary sodium intake produces a marked increase in Cyp11b2 (52). Instead, Cyp11b1 is constitutively expressed in zF cells and converts any available substrate into corticosterone (52), including any excess cholesterol that can be present in *Abcg1 cKO* adrenals. Therefore, we expect that the local concentration of cholesterol and steroid precursors may not affect aldosterone production.

Finally, although the extent to which our findings in mice are relevant to human pathophysiology remains to be explored, our data introduce the possibility that *Abcg1* variants may account for physiological or subclinical variation in stress response among healthy subjects. The Human Gene Mutation Database lists 25 different mutations or polymorphisms that have been described in *ABCG1* having a possible or probable pathological outcome (53-60). The individuals carrying these variants present with a series of phenotypes or risk associations predominantly linked to cardiovascular disorders, including impaired HDL homeostasis and increased risk for coronary heart disease. However, steroidogenic capacity in these individuals has not been assessed. Given the association between higher serum cortisol concentrations and cardiovascular risk profile (61), it would be of interest to assess basal and stimulated glucocorticoid levels in individuals carrying these alleles, which might explain interindividual variability in basal cortisol or physiological cortisol responses, and excess cortisol levels in individuals carrying risk alleles.

## Data Availability

Some or all datasets generated during and/or analyzed during the current study are not publicly available but are available from the corresponding author on reasonable request.
